# Headache as the Sole Symptom of Nasopharyngeal Carcinoma and Its Clinical Implications

**DOI:** 10.1100/2012/143829

**Published:** 2012-12-05

**Authors:** Yi-Lun Lee, Ching-Yin Ho

**Affiliations:** ^1^Department of Otolaryngology, Taipei Veterans General Hospital, No. 201, Section 2, Shih-Pai Road, Taipei 11217, Taiwan; ^2^School of Medicine, National Yang Ming University, Taipei 11217, Taiwan

## Abstract

*Background*. This study aimed to investigate the clinical features of NPC presenting with headache as the primary or sole symptom. *Methods*. The authors retrospectively identified 14 cases of NPC with headache as the initial presentation between 2003 and 2008. Headache characteristics, tumor staging, and treatment outcomes were assessed. *Results*. Most patients had either T4 (*n* = 12) or T3 (*n* = 1) tumor. The average duration of headaches prior to NPC diagnosis was 7.9 months. The location of the headaches was most commonly described as temporal or parietal with various pain patterns. Six patients (43%) experienced unilateral headache during attacks while the remaining patients reported bilateral or diffuse pain. Of the 14 patients, 10 (71%) experienced significant improvement in head pain during or after the treatment; most of them reported relief shortly after chemoradiation was initiated. The 5-year overall survival of these patients was similar to that of other NPC patients. *Conclusion*. Headache can be the only symptom of NPC. A timely diagnosis, albeit challenging to physicians, provides good outcomes in terms of both pain relief and tumor control.

## 1. Introduction

Nasopharyngeal carcinoma (NPC) is an Epstein-Barr virus (EBV)-related epithelial cancer that most commonly evolved from the pharyngeal recess posteromedial to the medial crura of the eustachian tube opening in the nasopharynx [1]. Although NPC is an uncommon malignancy in most parts of the world, it is endemic to certain well-defined populations. The highest incidence of NPC is found in a Cantonese region of South China around the city of Guangzhou, where the frequency of this disease is approximately 100-fold higher than in European and North American populations [2]. In Taiwan, there were 1579 new cases of NPC in the year 2007, and this condition ranked as the tenth most common cancer affecting the male population with an annual incidence rate of 10.05 per 100,000 male persons [3].

Headache is one of the various presenting symptoms of NPC, and its presence often indicates a skull base lesion or intracranial tumor invasion. Accordingly, this symptom hallmarks an advanced stage of the disease and implies poor prognosis. Although headache could be observed in up to 35%–50% of patients with NPC as an associated symptom, the number of those reporting headache as their initial or sole symptom is substantially lower [4, 5]. Nevertheless, headache is one of the most common complaints causing patients to call for medical help, and it has a lifetime prevalence of greater than 90% [6]. Regarding the scarcity of information about only headache in NPC, it is not uncommon for neurologists or primary care physicians to overlook the diagnosis of NPC in patients complaining of headache and without typical presentation of NPC, such as neck mass or eustachian tube-related symptoms.

This study aimed to describe the characteristics of a series of NPC patients who presented with headache as their initial or only symptom, to identify the warning features in such patients, and to investigate the treatment outcome of NPC in this specific subgroup.

## 2. Materials and Methods

### 2.1. Study Population

The Institutional Review Board at the Taipei Veterans General Hospital approved the study protocol. We used chart review to identify 14 patients visiting this tertiary referral center from 2003 to 2008 who presented with headache as their primary symptom and had been subsequently diagnosed as having NPC. The inclusion criteria for this patient group were as follows: (1) headache was the major or initial presenting symptom, (2) the diagnosis of NPC was obtained by pathological confirmation and complete staging workup, (3) no other definite secondary cause accounted for the symptom of headache, and (4) a complete record of treatment and followup was available. Patients with NPC who reported headache as an associated or minor symptom at the time of diagnosis were excluded from this survey. 

### 2.2. Interventions

All the patients were evaluated with a complete head and neck examination, including nasopharyngoscopy and biopsy of the lesion. The patients were asked to describe in detail the nature of their headaches and any associated symptoms. Staging investigations, including MRI of the nasopharynx, chest X-ray, abdominal sonography, whole-body bone scan, and/or whole-body positron emission tomography-computed tomography (PET-CT), were implemented.

 Chemoradiation plus induction and/or adjuvant chemotherapy were administered as the curative treatment for the patients with nonmetastatic disease. It comprised intensity-modulated radiation therapy (IMRT) with a total dose of 70 Gy administered in 35 fractions and cisplatin-based chemotherapy. All the patients received followup on a regular basis and had postradiation MRI checkups to examine the response to treatment at 3 months after the completion of IMRT. Disease- or treatment-associated symptoms were documented for subsequent systematic analysis.

### 2.3. Statistical Analysis

The follow-up period was measured from the day of the pathological confirmation of diagnosis to the day of death or the last clinic visit before analysis. Statistical Package for the Social Sciences (version 17, SPSS, Inc., Chicago, IL, USA) was used for the analysis. The actuarial overall survival was calculated by the Kaplan-Meier method.

## 3. Results

### 3.1. Patient Characteristics

We identified 256 cases of newly diagnosed NPC during the period of investigation in the Cancer Registry of Taipei Veterans General Hospital. Of these, 44 patients had reported headache as one of the presenting symptoms at the time of diagnosis. Fourteen patients fulfilled the inclusion criteria described above, with headache as the initial or main symptom of NPC, with 10 men and 4 women. Demographic data and the clinical parameters of these patients are presented in Table 1. The mean age of the patients was 55.4 years (range, 37–78 years). Preliminary neurological diagnoses of the 14 patients included the following conditions: migraine (*n* = 3), tension headache (*n* = 4), cluster headache (*n* = 2), neurosis (*n* = 1), and nonspecific headache (*n* = 4).

 Among the 14 enrolled patients, 10 were referred to the ENT clinic by neurologists after their brain imaging, magnetic resonance imaging (MRI) in most cases, showed a suspected nasopharyngeal or skull base lesion (Table 1). Furthermore, 3 of the 10 referred patients had an initial screening computed tomography (CT) scan of the brain that failed to detect the nasopharyngeal lesion due to insufficient lower edge setting or indistinct delineation of the skull base. Another 2 cases were immediately referred to us via private practitioners for further examination under an impression of secondary headache. The remaining two were accidentally found to have nasopharyngeal lesion in health checkups and were referred for further management. Both these patients had a history of newly onset headache and had been taking over-the-counter medications for pain relief.

### 3.2. Local Extension of Tumors

**Figure 1:**
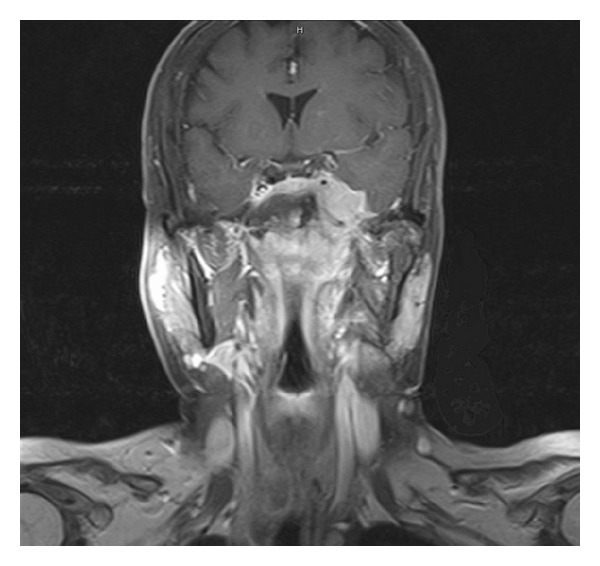
A representative case of ascending-type NPC. Coronal spin echo-enhanced T1-weighted MR image shows nasopharyngeal tumor with upward invasion into left cavernous sinus and medial temporal lobe. Small subclinical lymph nodes are noted at left neck.

The clinical staging was confirmed by MRI and/or PET-CT in a multidisciplinary NPC conference according to the 2002 American Joint Committee on Cancer (AJCC) staging system [7]. The local extent of the tumor in these 14 NPC patients was classified as T4 (*n* = 12), T3 (*n* = 1), and T1 (*n* = 1). Although physical examination of all the 14 patients who presented to our clinic showed no detectable neck node disease, 8 had occult nodal involvement, which was disclosed by MRI (Figure 1).

### 3.3. Pain Character and Associated Symptoms

Table 2 demonstrates the features of headaches. The location of the headaches varied. The headaches were described as temporal in 6 patients, parietal in 6, frontal in 2, and diffuse or unable to specify in 4 patients. The pain was pressing/tightening in quality in 4 patients, throbbing in 3, dull in 3, and explosive or debilitating in 3 patients. Six patients (43%) experienced unilateral headache during attacks while 3 (21%) definitively reported bilateral headache. The remaining 5 patients (36%) described the pain as diffuse or generalized. Although the mean duration of headache prior to the diagnosis of NPC was 7.9 months (range, 1–24 months), majority of the patients (9 out of 14; 64%) had been suffering from the symptom for less than 6 months, and 6 of them even experienced “acute” headaches that developed for less than 3 months. In most of the patients, the pain did not respond well to medications; therefore, brain imaging was arranged accordingly.

 The associated symptoms accompanying the headache attacks included facial numbness (*n* = 3), aural fullness/otalgia or tinnitus (*n* = 4), nasal congestion/rhinorrhoea (*n* = 2), blurred or double vision (*n* = 5), tearing (*n* = 1), sweating (*n* = 1), phonophobia (*n* = 1), and ptosis (*n* = 1).

### 3.4. Treatment Outcomes

The summary of treatment modalities and follow-up status is demonstrated in Table 1. Two patients (patients 6 and 8) received palliative treatment due to the systemic involvement of NPC and poor medical condition, respectively. Among the 12 patients who received curative treatment, one (patient 7) expired due to complications of aspiration pneumonia 10 months after the completion of chemoradiation plus adjuvant CT, with a persistent disease noted on the follow-up MRI. Another patient (patient 10), who suffered from distant failure with good locoregional control, was alive with disease and available for regular clinic followups. The remainder of the patients (10 of 12, 83%) was clinically stable, either with complete or partial response, without interval changes in the skull base lesion on follow-up MRI. The mean duration of the followup was 25 months (range, 4–61 months). We compared the cumulative survival function of the 14 patients (with headache as the only symptom) and of the remaining 242 patients with NPC and found that the overall survival was slightly worse at 78% in the first group at the 2-year followup; however, this difference was statistically insignificant at the 5-year followup.

Of the 14 patients, 10 (71%) experienced significant improvement in head pain during or after the treatment; most of them reported relief shortly after chemoradiation was initiated. We could not find any definitive description of headache relief on the chart records of the rest of the patients; however, none of them complained of headache or used analgesics for pain relief at the follow-up visits.

 In general, these patients responded fairly well to the treatment in terms of both tumor control and headache relief by an adequate follow-up period.

## 4. Discussion

The content of this manuscript should serve as a reminder to physicians who treat headaches to be aware of the possibility that recurrent or persistent headaches could be the most prominent manifestation of NPC or other skull base pathologies and that a routine brain CT scan for screening the intracranial lesion could miss a subtle lesion at the skull base. In our study, among the 10 patients who were referred from neurologists, NPC was almost exclusively diagnosed from brain MRI (8 of 10 patients), including 3 patients with negative findings on their previous brain CT scans. Thus, clinicians must understand that the routine setting of a noncontrast brain CT, which is frequently arranged for headache patients as a screening tool, is limited with regard to detecting possible skull base or nasopharyngeal lesions. 

It has been proposed that the advanced NPCs can be divided into three clinical types, namely, ascending type, descending type, and mixed type [8]. The ascending-type NPC extends directly toward the base of skull with frequent involvement of cranial nerves [9]. However, there is no cervical lymphadenopathy in this entity. Headache is the most prominent and often the first symptom in this situation, which represents a diagnostic challenge for clinicians. To facilitate early diagnosis, it is of great importance to recognize the headache pattern of ascending-type NPC; yet, to the best of our knowledge, there has never been any pertinent study addressing this issue. Our report is the first to analyze the characteristics of headache in these unique patients. 

It is difficult or can be impossible to distinguish an NPC-related headache from a primary one. The International Classification of Headache Disorders, 2nd edition (ICHD-II) contains extensive illustration of the diagnostic criteria of primary headaches [10]. In typical cases, diagnosis is not particularly difficult as it might be when one or more of the main features required by the classification is missing, or when other atypical elements are present. On the other hand, it is possible that clinical features, which at the beginning are virtually indistinguishable from a primary headache, may later be proved to be related to a secondary cause: this is true not only for tension-type headaches due to their relatively nonspecific features but also for primary headaches possessing more specific characteristics such as migraine and cluster headache [11]. Accordingly, in clinical practice, one could possibly categorize a patient with NPC presenting with headache as the main manifestation as a victim of primary headache. A recent comprehensive review of cluster-like headaches (CLHs) revealed that neoplasm is the second most frequent pathology in association with CLHs, accounting for 25.7% of CLH patients, following vascular lesions [11]. In our series, 3 patients (21%) who initially had only headache later developed ptosis and/or facial numbness, and CLH was suspected in two of them as the preliminary diagnosis by neurologists. 

 It must be noted that the pathognomonic features of secondary headache attributed to NPC are still inconclusive for the limited size of our survey. Nonetheless, a general principle still stands out: if a headache patient failed to fulfill the complete diagnostic criteria of primary headaches, further investigation to exclude a secondary cause is indispensable, and brain MRI would be more effective for this purpose than CT scan.

Chemoradiation resulted in very good improvement in the NPC-related headaches in our series. For these patients, the headache symptom was greatly reduced or resolved in close temporal relation to the chemotherapy and/or radiotherapy treatment administered. Although most of the patients with NPC having headache as the primary presentation had advanced disease at the time of diagnosis, the prognosis regarding the overall survival did not appear compromised in these patients as compared to general NPC patients if appropriate treatment was administered.

## 5. Conclusion

NPC plays a role in the differential diagnosis of headache, especially in the endemic countries, and a correct diagnosis is challenging in patients without typical presentations. There are no definitive clinical features of NPC-related headaches, and negative brain CT scans do not necessarily exclude this diagnosis. Patients with primary headaches who do not respond to treatment may benefit from consultation with an otolaryngologist. Readily available fiberoptic nasopharyngoscopy should be considered before further brain imaging is undertaken, as it might provide additional information in this situation. Relief from headache and appropriate tumor control of this NPC subgroup could be expected by treatment with chemoradiation.

## Figures and Tables

**Table 1:** Characteristics of NPC patients with headache as the initial presentation.

Pt.	Age	Sex	T staging	Diagnostic image	Treatment	Followup (mo)	Remission of headache
1	40	M	T4	MR	IC/CCRT/AC	22/AWD	N/A
2	55	M	T4	MR	IC/CCRT	61/NED	Y
3	52	M	T4	MR	IC/CCRT/AC	53/NED	Y
4	78	M	T4	CT	IC/CCRT/AC	20/PF	Y
5	64	F	T1	—	RT alone	44/NED	Y
6	50	M	T4	MR	Palliative RT	19/expired	N/A
7	53	M	T4	—	IC/CCRT/AC	17/expired	N/A
8	77	M	T4	MR	Palliative RT	4/expired	N/A
9	69	M	T4	MR	IC/CCRT	15/PF	Y
10	43	F	T4	MR	IC/CCRT/AC	30/DM	Y
11	61	F	T4	MR	IC/CCRT/AC	20/PF	Y
12	48	M	T3	—	CCRT	15/NED	Y
13	49	F	T4	—	IC/CCRT/AC	14/NED	Y
14	37	M	T4	CT	IC/CCRT/AC	24/NED	Y

NED: no evidence of disease, PF: progression-free, DM: distant metastasis, AWD: alive with disease.

**Table 2:** Features of the headaches.

Patient	Duration prior to NPC diagnosis (months)	Pain distribution	Pain quality	Associated or late-onset symptoms
1	3	Left temporal, radiating to parietal area	Pressure-like	Aural fullness/tinnitus
2	18	Bilateral temporal, occasionally left frontal	Dull	—
3	6	Diffuse	Nonspecific	Otalgia
4	5	Left parietal	Dull	Tinnitus/tearing
5	12	Bilateral temporal	Dull	Rhinorrhea
6	5	Right parietal, radiating to vertex	Pulsating	Phonophobia/sweating/diplopia
7	2	Frontal	Explosive	Nasal obstruction/diplopia
8	12	Diffuse, involving nuchal region	Pulsating	—
9	2	Diffuse	Pressing	Facial numbness/ptosis
10	18	Bilateral temporal	Tightening	Facial numbness
11	24	Right parietal	Pressing	Blurred vision
12	1	Left temporal, radiating to parietal area	Pulsating	Aural fullness/otalgia/tinnitus
13	2	Left temporal, radiating to parietal area	Debilitating	Facial numbness/diplopia
14	2	Diffuse	Explosive	Diplopia

## References

[1] Sham JST, Wei WI, Yong-Sheng Z (1990). Detection of subclinical nasopharyngeal carcinoma by fibreoptic endoscopy and multiple biopsy. *The Lancet*.

[2] Chang ET, Adami HO (2006). The enigmatic epidemiology of nasopharyngeal carcinoma. *Cancer Epidemiology Biomarkers and Prevention*.

[4] Lee AW, Foo W, Law SC (1997). Nasopharyngeal carcinoma: presenting symptoms and duration before diagnosis. *Hong Kong Medical Journal*.

[5] Hsu MM, Tu SM (1983). Nasopharyngeal carcinoma in Taiwan. Clinical manifestations and results of therapy. *Cancer*.

[6] Rasmussen BK, Jensen R, Schroll M, Olesen J (1991). Epidemiology of headache in a general population—a prevalence study. *Journal of Clinical Epidemiology*.

[7] Greene FL, Page DL, Fleming ID (2002). *AJCC Cancer Staging Manualed*.

[8] Hsieh CK, Li CC, Min HC, Chang FL, Li PK (1965). Clinical analysis of 1,000 cases of nasopharyngeal carcinoma with particular reference to early diagnosis and clinical types of late cases. *Chinese Medical Journal*.

[9] Chen MS, Lin FJ, Tang SG, Leung WM, Leung W (1989). Clinical significance of cranial nerve deficit in the therapy of nasopharyngeal carcinoma. *British Journal of Radiology*.

[10] (2004). The International Classification of Headache Disorders: 2nd edition. *Cephalalgia*.

[11] Mainardi F, Trucco M, Maggioni F, Palestini C, Dainese F, Zanchin G (2010). Cluster-like headache. A comprehensive reappraisal. *Cephalalgia*.

